# Effect of the Gupta Score on Pre-operative Cardiology Consultation Requests in Noncardiac Nonvascular Surgery

**DOI:** 10.4274/TJAR.2023.231464

**Published:** 2023-12-27

**Authors:** Funda Atar, Fatma Özkan Sipahioğlu, Gülsen Keskin, Aslı Dönmez

**Affiliations:** 1 University of Health Sciences Turkey, Etlik City Hospital, Clinic of Anaesthesiology and Reanimation, Ankara, Turkey

**Keywords:** Cardiac risk stratification, cardiology consultation, pre-operative care

## Abstract

**Objective:**

Cardiologists are the most frequently consulted specialists during pre-operative evaluations. However, unnecessary cardiology consultations (CC) can increase cardiologists’ workload without impacting anaesthesia practice, resulting in delayed surgeries and additional financial burdens. We hypothesize that using Gupta during the preoperative period can reduce these adverse effects.

**Methods:**

This prospective study included patients scheduled for elective noncardiac, nonvascular surgeries who underwent pre-operative assessment. Patients who had no specific risk index used for preoperative cardiac risk evaluation were classified as Group I, and those evaluated using the Gupta scale were classified as Group II. The study compared preoperative CC, diagnostic tests, surgical delays, major adverse cardiac event (MACE), length of hospital stay and intensive care unit (ICU) stay, mortality, and costs.

**Results:**

A total of 898 patients were included in the study, with 487 in Group I and 411 in Group II. The Gupta group reduced the demand for preoperative CC (P<0.001) and preoperative non-invasive diagnostic testing (n = 107, 21.9% vs. n = 36, 8.75%). The time from the anaesthesiology outpatient clinic to surgery was 15 days in Group I and 14 days in Group II (*P*=0.132). The length of ICU stay was higher in Group I (*P*=0.019). MACE was 15 patients (3.08%) in Group I and 9 patients (2.19%) in Group II (*P*=0.076). The cost of patients in Group I was higher than that in Group II (*P*=0.019).

**Conclusion:**

Using Gupta in preoperative evaluation may reduce unnecessary preoperative resource usage, surgical delays, ICU hospitalization rates, additional costs, and mortality.

**Main Points**

• It was determined that using the Gupta score before elective noncardiac, nonvascular surgery decreased preoperative cardiology consultation and non-invasive diagnostic tests.

• It was observed that the time to surgery and the length of stay in the intensive care unit decreased in patients who were evaluated by using the Gupta score.

• Changing the perspective on preoperative cardiology consultation and requesting more rational consultations may be cost-effective.

## Introduction

As 42% of overall complications in noncardiac, nonvascular surgery (NCNVS) stem from cardiac-related issues, cardiologists are the most commonly consulted specialists during pre-operative evaluations.^1^ However, unnecessary cardiology consultations (CCs) can increase cardiologists’ workload without impacting anaesthesia practice, resulting in delayed surgeries, wasted time, and additional financial burdens.^2^ Recently, a predictive model called the Gupta score was developed, which uses the American College of Surgeons National Surgical Quality Improvement Program (NSQIP) database to estimate the risk of perioperative major adverse cardiac events (MACEs), such as myocardial infarction (MI) or cardiac arrest.^3^ The Gupta score is an interactive risk calculation program.^3^ The risk score comprises 5 items: type of surgery, the participant’s functional status, abnormal creatinine levels (>130 mmol L or >1.5 mg dL^-1^), American Society of Anesthesiologists (ASA) classification, and age.^4^ Unlike previously used indexes, the Gupta score provides individualized probability estimation for MACE rather than a scoring system. Based on the Gupta score, patients with an estimated perioperative MACE risk of <1% can proceed with surgery without requiring further cardiac workup, whereas those with a risk of MACE exceeding 1% are considered high-risk and may necessitate CC for preoperative testing and treatment.

Although the surgical risk models suggested by the current guidelines recommend avoiding unnecessary preoperative consultation and workup, the effect of these risk models on the CC rate and optimal preoperative evaluation is not obvious in daily practice. This study aims to address this gap by evaluating the effect of the Gupta score on the CC rate in patients scheduled for elective, intermediate/high-risk NCNVS. Additionally, the study seeks to observe the broader impact of implementing a strategy based on the Gupta score on perioperative clinical outcomes, resource utilization [including transthoracic echocardiography (ECO), Holter monitorization, scintigraphy, coronary angiography, etc.], and additional costs.

## Methods

The study was approved by the Clinical Research Ethics Committee of University of Health Sciences Turkey, Dışkapı Yıldırım Beyazıt Training and Research Hospital (approval no: 128/21, date: 10.01.2022), and this trial was registered at ClinicalTrials.gov (NCT05532917). Written informed consent was obtained from all patients participating in the trial. Informed consent was obtained from each patient, and the study protocol conformed to the ethical guidelines of the 1975 Declaration of Helsinki as reflected in a priori approval by the institution’s human research committee.

From February 01, 2022 to March 31, 2022, patients aged ≥18 years who were scheduled for elective intermediate/high-risk NCNVS underwent routine preoperative assessment in an outpatient clinic. The type of surgery was categorized on the basis of surgical risk, following the American College of Cardiology/American Heart Association classification.^5^ Each patient received a comprehensive evaluation, including medical history, physical examination, electrocardiogram, complete blood cell count, chemistry, chest roentgenogram, and any additional assessments deemed necessary by the anaesthesiologist.

The ASA classification was used as an index to determine a patient’s general status.^2^ The New York Heart Association Functional Classification (NYHA) and Revised Cardiac Risk Index (RCRI) were calculated for each patient.^6^ The study population was divided into 2 groups based on their preoperative cardiac risk assessment: Group I (no specific risk index used for preoperative cardiac risk evaluation) and Group II (using the Gupta score for preoperative cardiac risk assessment). Two different expert anaesthetists performed the pre-operative assessment.

The main reason for referral for CC was classified into 8 categories: a. hypertension (HT), b. general evaluation, c. coronary artery disease (CAD)- anticoagulation management, d. elderly patient, and e. electrocardiography (ECG) changes, f. valve abnormality, and h. other. Demographic and personal characteristics of patients [ASA, age, gender, body mass index, time to CC and surgery, length of stay hospital and intensive care unit (ICU), the surgery type and risk, diagnostic tests requested by the cardiologist (ECG, ECO, Holter monitoring, cardiovascular stress test, scintigraphy, coronary angiogram, percutaneous coronary intervention (PCI)], NYHA, RCRI, 30-day mortality, and MACE were recorded. MACE was defined according to the NSQIP: Documentation of ECG changes indicative of acute MI (one or more of the following: ST-elevation >1 mm in 2 or more contiguous leads, new left bundle branch block, new q‐wave in 2 or more contiguous leads); new elevation in troponin greater than 3 times the upper level of the reference range in the setting of suspected myocardial ischemia.^7^ The cost was determined by scanning accessible data in hospital billing statements and calculating charges for each test ordered and the hospitalization.

The data were statistically analyzed using IBM SPSS Statistics for Windows, Version 20.0. package program. Data are summarized as mean ± standard deviation and median (25-75%) for continuous variables, frequencies, and percentiles for categorical variables. The Mann-Whitney U test and Student’s t-test were used for independent group (Group I, n = 487 and Group II, n = 411) comparisons, depending on the distributional properties of the data based on groups (according to results of Shapiro Wilk test). The chi-square test was used for proportions, and its counterpart Fisher’s exact test was used when the data were sparse. For all statistical analyses, any *P* value less than 0.05 was considered statistically significant.

## Results

**Figure 1:**
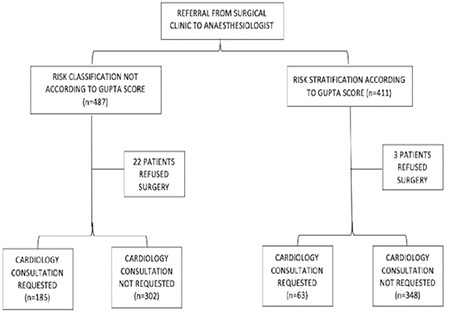
Flowchart of the study

A total of 898 patients were included in the study, with 487 in Group I and 411 in Group II. During the pre-operative period, 22 (4.52%) patients in Group I and 3 (0.73%) patients in Group II refused surgery (*P*=0.001) (Figure 1). Preoperative CC was performed by 185 (37.9%) patients in Group I and 63 (15.3%) patients in Group II (*P* < 0.001). Demographic data, ASA, NYHA, comorbidity, and RCRI were similar in both groups. The smoking rate was higher in Group II (Table 1). In Group I, the most common reasons for consultation were HT (n = 44, 23.78%) and general evaluation (n = 37, 20%). The mean age of Group I was 55.57±16.06 years, whereas for patients who requested CC due to the general evaluation, the mean age was 62.05±9.03 years. Other preoperative reasons leading to consultation with a cardiologist are listed in Table 2.

**Figure 2:**
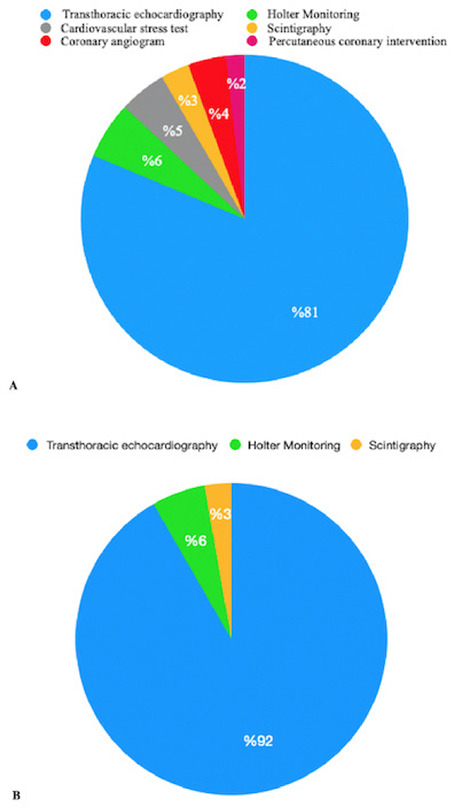
Tests ordered in patients with cardiology consultation. A: in Group I, B: in Group II

Preoperative cardiac testing was more common in Group I patients than in Group II patients (n = 107, 21.9% vs n = 36, 8.75%). ECO was the most frequently performed test in both groups (n = 87, 60% in Group I; n = 33, 23.07% in Group II; *P* < 0.01, respectively), followed by Holter monitoring in 8 cases (5.6%). In Group I, other performed tests included exercise stress ECG (n = 5, 4.6%), coronary angiogram (n = 4, 3.7%), myocardial scintigraphy (n = 3, 2.8%), and PCI (n = 2, 1.8%). None of the patients in Group II requested cardiovascular stress testing, angiography, scintigraphy, or PCI (Figure 2).

In both groups, patients who requested CC were frequently examined by a cardiologist in the outpatient anaesthesiology  clinic on the same day [interquartile range (IQR) 0-1]. The time interval from the anaesthesiology  outpatient clinic to surgery was 15 days (IQR 7-31) in Group I and 14 days (IQR 7-28) in Group II (*P*=0.132). A total of 15 patients (3.08%) in Group I and 9 patients (2.19%) in Group II had perioperative cardiovascular complications (p= 0.076). The distribution of cardiovascular complications was comparable between the two groups (*P*=0.14). The most common cardiac complication was acute coronary syndrome (Table 3).

The hospital length of stay for the patients was similar between the two groups (*P*=0.385), whereas the ICU length of stay was higher in Group I (3.88±4.55 vs. 2.47±2.44, *P*=0.019). The 30-day mortality rate was 2.26% (n = 11) in Group I and 0.97% (n = 4) in Group II (*P*=0.191). The cost of patients in Group I was higher than that in Group II 63.0 (43.0-566651.20) TL vs. 53.13 (22.52-56570.0) TL, *P*=0.019) (Table 3).

## Discussion

The present study shows that using the Gupta score before elective NCNVS reduces preoperative CC. Furthermore, there was a decrease in the number of preoperative non-invasive diagnostic tests requested when the Gupta score was used. In patients who used the Gupta score, the time to surgery decreased by approximately 1 day, and the length of stay in the intensive care unit decreased by an average of 1.41 days. Although there was no statistical difference, adhering to the Gupta score resulted in fewer occurrences of MACE. Moreover, the use of the Gupta score for the desired CC was found to be more cost-effective.

Preoperative cardiac evaluation based on guidelines has significantly reduced unnecessary consultations.^8,9^ Kleinman et al.^10^ argued that CC requests were necessary and could detect newly diagnosed HT and angina in 15% of the study groups. However, the detection of any clinical problem by cardiologists contributed little to clinical decision-making and did not reduce perioperative cardiovascular complications.^11^ The fear of missing important issues or malpractise lawsuits might have led clinicians to lower the threshold for requesting preoperative consultations. Nevertheless, most consultations provide no suggestions beyond “cleared for surgery”, “proceed with the case”, or “continue present medications”.^11^ Demand for preoperative consultations based on cardiac risk indices may reduce unnecessary investigations, improve cost-effectiveness, and avoid delays. We observed that adhering to the Gupta score for cardiac evaluation before NCNVS reduced the incidence of preoperative CC by more than half. Consequently, following and applying current risk models can help reduce unnecessary consultations.

In our study, among patients who did not undergo a specific protocol for preoperative cardiovascular evaluation, HT and general evaluation were the most common causes of CC. It has been observed that controlled HT may cause unnecessary CC because it does not affect cardiovascular morbidity or mortality.^12^ Therefore, HT alone may not be a sufficient reason for consultation. Another probable issue is that the physician initiating the consultation might not have clearly communicated to the cardiologist why the consultation is being sought. We found that the mean age of the patients who were requested to undergo CC due to the general evaluation in our study was 62 years, which may have contributed to this higher rate. However, this non-specific manner of referral often leads to a general diagnostic work-up and reduces the impact of CC on perioperative management.^11^ Based on these results, we predict that stating the indications for the consultation request correctly and clearly can reduce the unnecessary burden and waste of resources in the cardiology department.

The Gupta score reduced the use of preoperative non-invasive diagnostic tests. Furthermore, when CC was requested based on the Gupta score, there was a reduced need for ECO, and no requests were made for cardiovascular stress tests, angiography, scintigraphy, or PCI. Additionally, the time from the anaesthesia outpatient clinic to the surgery was approximately 24 h less in patients using the Gupta score. We believe that more appropriate and less demanding preoperative cardiac tests may cause this situation. Similarly, excessive preoperative cardiac testing can cause surgery delays and increase mortality during the perioperative period.^11,12,13^ We acknowledge that unnecessary CC requests and preoperative cardiac tests are not the only factors causing the delay; it may be multifactorial. However, it has been shown that minimizing surgical delay can reduce mortality.^14^ Therefore, we believe that it is necessary to weigh the benefits of further cardiac evaluation for preoperative optimization versus the morbidity and mortality caused by the delay in surgery.

CAD can be considered one of the most critical comorbidities expected to increase the risk of perioperative MACE.^9^ In our study population, both groups scheduled for intermediate/high-risk surgical procedures had multiple risk factors for CAD or a history of ischemic heart disease. Clinicians may have demonstrated an increased tendency for CC in this patient group because of the perceived risk of perioperative MI and other significant adverse cardiac events.^9^ Nevertheless, although our study had no statistical difference, MACE was seen less frequently when adhering to the Gupta score. Routine cardiac examination for CAD assessment is not entirely safe and often does not contribute to preoperative clinical decision-making preoperatively.^15,16^ Therefore, current guidelines do not recommend routine preoperative CC for patients with CAD or risk factors.^6^ In conclusion, in patients with cardiac comorbidities, the desired CC based on current risk models appears to be more effective than an approach based on routine cardiac examination.

Our study demonstrated that the Gupta score for CC resulted in decreased resource usage (cardiac diagnostic test), leading to increased efficiency. This reduction in resource utilization alleviates the workload of healthcare staff and offers economic advantages. Approximately 20-34% of healthcare costs are spent on ineffective measures are indicated. Hence, identifying and mitigating these unnecessary expenses has become of paramount importance. Cost-effective healthcare delivery is especially crucial for developing countries. One of the major contributors to healthcare costs is the inappropriate use of advanced medical technology and services.^17^ Non-specific consultations and workups may lead to false positive results, unnecessary, costly, and potentially harmful treatments, or further evaluation that may delay surgery.^18^ If the findings of our study were generalized to other clinics nationwide, we believe it could substantially reduce unnecessary costs.

## Conclusion

Several remarks must be considered when interpreting these results. Despite the completeness of the collected data and the high level of follow-up, the study could not be randomized. In addition, surgery delay is multifactorial, and other relevant factors were not included in our analysis.

In conclusion, the Gupta score enables patients to easily and accurately calculate their preoperative mortality risk at the bedside or in the clinic. Thus, unnecessary consultations, workups, surgery delays, and additional costs can be avoided.

## Figures and Tables

**Table 1:**
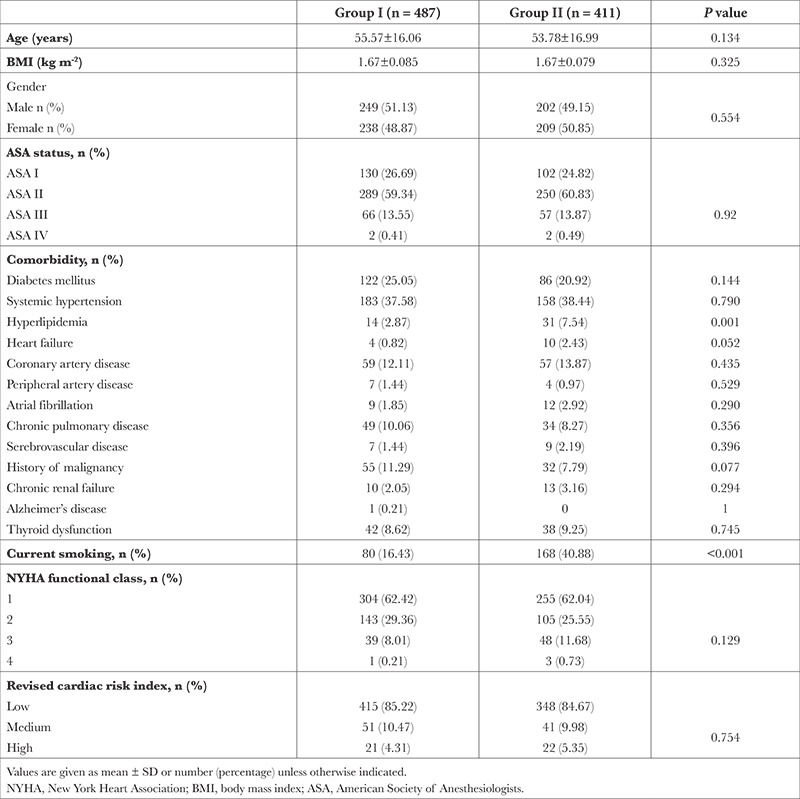
Demographic and Clinical Characteristics, NYHA Functional Class and Revised Cardiac Risk Index of Patients

**Table 2:**
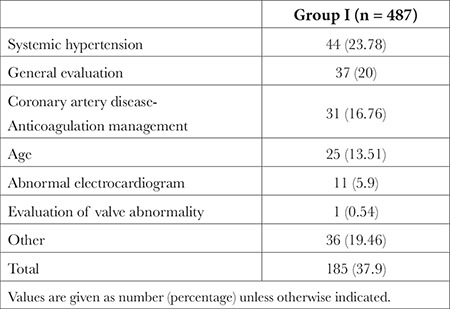
Main Reason to Refer a Patient to a Cardiologist in Group I

**Table 3:**
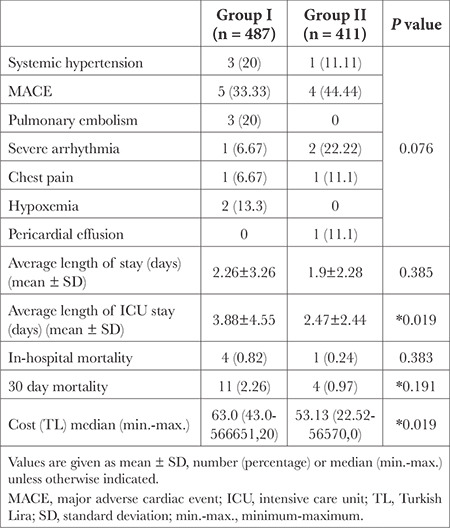
Adverse Perioperatif Cardiovascular and Noncardiovascular Outcomes
